# Multidimensional effects of a school-based martial arts intervention on physical fitness, executive function, and psychological resilience in adolescents: a quasi-experimental study

**DOI:** 10.3389/fpsyg.2026.1891636

**Published:** 2026-10-23

**Authors:** Yuanyuan Ma, Jun Liang

**Affiliations:** 1 Henan Finance University, Zhengzhou, Henan Province, China; 2 Nanchang Institute of Technology, Nanchang, Jiangxi, China

**Keywords:** adolescent health, adolescents, executive function, martial arts, perceived stress, physical fitness, psychological resilience, self-regulation

## Abstract

**Background:**

Adolescence is a critical developmental period during which physical fitness, executive function, and psychological resilience are closely interconnected. School-based martial arts training may provide a multidimensional intervention approach by combining physical exercise, cognitive engagement, self-regulation, and psychosocial development. This study examined the effects of a 12-week school-based martial arts intervention on physical fitness, executive function, psychological resilience, self-regulation, and perceived stress among adolescents.

**Methods:**

A quasi-experimental repeated-measures design was adopted. A total of 120 adolescents aged 13–16 years from three middle schools in Nanchang, China, were allocated into three groups: School-Based Martial Arts Training Group (SMATG; *n* = 40), Traditional Physical Education Group (TPEG; *n* = 40), and Control Group (CG; *n* = 40). The intervention was conducted three times per week for 60 min per session over 12 weeks. Outcome variables included agility, dynamic balance, muscular power, cardiorespiratory endurance, inhibitory control, working memory, cognitive flexibility, psychological resilience, self-regulation, and perceived stress. Assessments were conducted at baseline, 6 weeks, and 12 weeks. Data were analyzed using Mixed MANOVA and Bonferroni-adjusted *post hoc* comparisons.

**Results:**

Significant Group × Time interaction effects were found across all physical, cognitive, and psychological outcomes (*p* < 0.001). Compared with the TPEG and CG, the SMATG showed greater improvements in agility, dynamic balance, standing broad jump performance, Estimated VO₂peak, inhibitory control, working memory, cognitive flexibility, psychological resilience, and self-regulation, as well as greater reductions in perceived stress. Moderate-to-large effect sizes were observed across multidimensional outcomes.

**Conclusion:**

The findings suggest that structured school-based martial arts training may be a promising approach for supporting multiple physical, cognitive, and psychological outcomes in adolescents.

## Introduction

Adolescence is a pivotal developmental window during which physical fitness, cognitive performance, and psychological resilience undergo rapid and interdependent changes. Despite the well-established health benefits of regular physical activity, global surveillance data from a pooled analysis of 298 population-based surveys, representing over 1.6 million participants, revealed that more than 80% of school-going adolescents aged 11–17 years do not meet the World Health Organization’s recommended minimum of 60 min of moderate-to-vigorous physical activity per day (Guthold et al., 2020). This pervasive physical inactivity is accompanied by deteriorating trends in cardiorespiratory fitness, muscular strength, and motor competence among youth worldwide (Habyarimana et al., 2025). Declining physical fitness in adolescence is not merely a cardiometabolic concern; it is closely linked to impaired cognitive development, reduced academic performance, and heightened psychological vulnerability (Lubans et al., 2016), underscoring the urgent need for effective, evidence-based school-based physical activity interventions.

Among the cognitive domains most sensitive to physical activity, executive function, encompassing inhibitory control, working memory, and cognitive flexibility, occupies a central role in adolescent learning, behavioral self-regulation, and adaptive functioning (Li X. et al., 2024). Converging evidence from systematic reviews and meta-analyses demonstrates that structured physical activity interventions, particularly those incorporating open-skill and cognitively demanding motor tasks, yield meaningful and consistent improvements in executive function outcomes in children and adolescents (Mao et al., 2024; Teng et al., 2025). A network meta-analysis of randomized controlled trials further identified cognitively engaging and coordinative physical activities as the most efficacious modalities for enhancing inhibitory control, working memory, and cognitive flexibility in youth populations (Wang et al., 2023).

Martial arts have attracted increasing scientific attention as a uniquely multidimensional physical activity modality capable of simultaneously targeting physical, cognitive, and psychosocial outcomes. Unlike conventional physical education, martial arts training integrates progressive technical skill acquisition, rule-based behavioral codes, attentional demands, rapid decision-making, and controlled movement sequencing, characteristics that generate both physiological and neuromotor stimulation (Harwood-Gross et al., 2021). The seminal work of Lakes and Hoyt (2004) demonstrated that school-based Taekwondo training significantly improved cognitive self-regulation, affective self-regulation, and prosocial behavior in elementary school children compared to traditional physical education. Subsequently, Pinto-Escalona et al. (2024) reported that a one-year school-based karate intervention delivered during regular physical education lessons improved academic achievement, conduct behavior, cardiorespiratory fitness, balance, and flexibility in children aged 7–8 years across five European countries. More recent experimental evidence from at-risk adolescent populations further confirmed that 6 months of martial arts training produced significant improvements in inhibitory control, cognitive flexibility, and self-esteem compared to standard sport participation (Harwood-Gross et al., 2021).

The psychological dimensions of martial arts training are equally compelling. Structured martial arts practice, emphasizing discipline, impulse control, goal pursuit, and coping under pressure, aligns directly with mechanisms underlying psychological resilience and self-regulation development (Li X. et al., 2024). Studies involving combat sports-based interventions have documented reductions in perceived stress, improved emotional regulation, and enhanced resilience in youth participants (Xu et al., 2025). Physical activity more broadly has been shown to foster adolescent resilience through the mediation of self-efficacy and basic psychological needs satisfaction (Li X. et al., 2024), while structured physical education programs that integrate cognitive challenge further amplify these psychological benefits (Li Z. et al., 2024; Zhang et al., 2024).

Despite growing evidence supporting the benefits of martial arts participation in youth populations, most previous investigations have focused on isolated physical, cognitive, or psychological outcomes independently, often using short intervention durations or single-group designs. Comprehensive school-based studies simultaneously examining physical fitness, executive function, and psychological resilience within a repeated-measures adolescent framework remain limited. Furthermore, direct comparison of a structured martial arts intervention with both traditional physical education and control condition conditions in a real-world educational setting has not been sufficiently established. Therefore, the present study aimed to examine the multidimensional effects of a 12-week school-based martial arts intervention on physical fitness, executive function, and psychological outcomes among adolescents using a quasi-experimental repeated-measures design. We hypothesized that participants in the SMATG would demonstrate greater improvements in physical fitness, executive function, and psychological outcomes than participants in the TPEG and CG at the mid- and post-intervention assessments.

## Materials and methods

### Participants of the study

A total of 120 male adolescent students (aged 13–16 years) were recruited from three middle schools in Nanchang using a quasi-experimental cluster sampling approach. Sex was therefore not treated as a between-participant variable in the present study, and no sex-stratified analyses were performed. The study was conducted over a period of 5 months, from September 2025 to January 2026. An *a priori* sample-size calculation was conducted using G*Power based on the planned individual-level repeated-measures analysis. The calculation indicated a minimum of 108 participants; 120 participants were recruited to allow for potential attrition. Because allocation occurred at the school level, this calculation does not account for potential intracluster correlation and should therefore be interpreted as an individual-level planning estimate. Participants were assigned into three groups: School-Based Martial Arts Training Group (SMATG; *n* = 40), Traditional Physical Education Group (TPEG; *n* = 40), and Control Group (CG; *n* = 40).

Because the intervention was implemented within existing school structures, allocation occurred at the school level rather than through individual randomization. Each participating school implemented one study condition: SMATG, TPEG, or CG. Consequently, school-level characteristics, including differences in the educational environment, school facilities, teaching practices, peer environment, and other contextual factors, may be correlated with the assigned intervention condition. Thus, the observed differences between groups cannot be interpreted as being attributable exclusively to the martial arts intervention, and a potential school-level confounding effect must be considered when interpreting the findings. Individual-level randomization was not feasible because of school administrative and scheduling constraints. The G*Power calculation was based on an individual-level statistical framework and therefore did not account for potential clustering or intraclass correlation associated with school-level allocation. All participants were regularly attending school physical education classes and were medically cleared for physical activity participation.

Inclusion criteria comprised: (i) adolescents aged between 13 and 16 years, (ii) enrollment in regular school physical education programs, and (iii) absence of any diagnosed neurological, cardiovascular, or musculoskeletal disorders. Exclusion criteria included: (i) history of severe injury within the previous 6 months, (ii) participation in organized martial arts training outside the school environment, and (iii) absence from more than 15% of intervention sessions during the study period. Written informed consent was obtained from both participants and their parents or legal guardians prior to participation. Participants were informed about the purpose, procedures, potential benefits, and risks of the study, and were assured of their right to withdraw at any stage without academic or personal consequences. The study protocol was conducted in accordance with the Declaration of Helsinki for research involving human participants.

Table 1 presents the descriptive characteristics of participants across the school-Based Martial Arts Training Group (SMATG), Traditional Physical Education Group (TPEG), and Control Group (CG). Baseline characteristics indicated that all groups were comparable in terms of age, height, weight, and body mass index (BMI), indicating baseline comparability among the study groups.

**Table 1:** Descriptive characteristics of participants across groups (mean ± standard deviation).

Variable	SMATG (*n* = 40)	TPEG (*n* = 40)	CG (*n* = 40)	*F*-value	*p*-value
Age (years)	14.52 ± 0.91	14.47 ± 0.88	14.49 ± 0.93	0.04	0.961
Height (cm)	160.41 ± 7.84	159.92 ± 8.02	160.16 ± 7.73	0.03	0.972
Weight (kg)	52.73 ± 6.81	53.11 ± 6.66	52.88 ± 6.74	0.04	0.958
BMI (kg/m^2^)	20.43 ± 2.18	20.71 ± 2.26	20.56 ± 2.20	0.12	0.887

### Study design

The present study employed a quasi-experimental repeated-measures design with three parallel conditions: a School-Based Martial Arts Training Group (SMATG), a Traditional Physical Education Group (TPEG), and a usual-activity Control Group (CG). The intervention period lasted 12 weeks, with assessments conducted at baseline, 6 weeks, and 12 weeks. Participants were recruited from three middle schools in Nanchang, China, and study conditions were implemented at the existing school/class level because individual randomization was not feasible within the participating educational settings. Consequently, each school implemented one study condition, creating potential confounding between school-level characteristics and intervention condition. The principal exposure variable was study condition, while outcomes comprised measures of physical fitness, executive function, psychological resilience, self-regulation, and perceived stress. The repeated-measures design was used to examine changes in outcomes across the three assessment points (see Figure 1).

**Figure 1:**
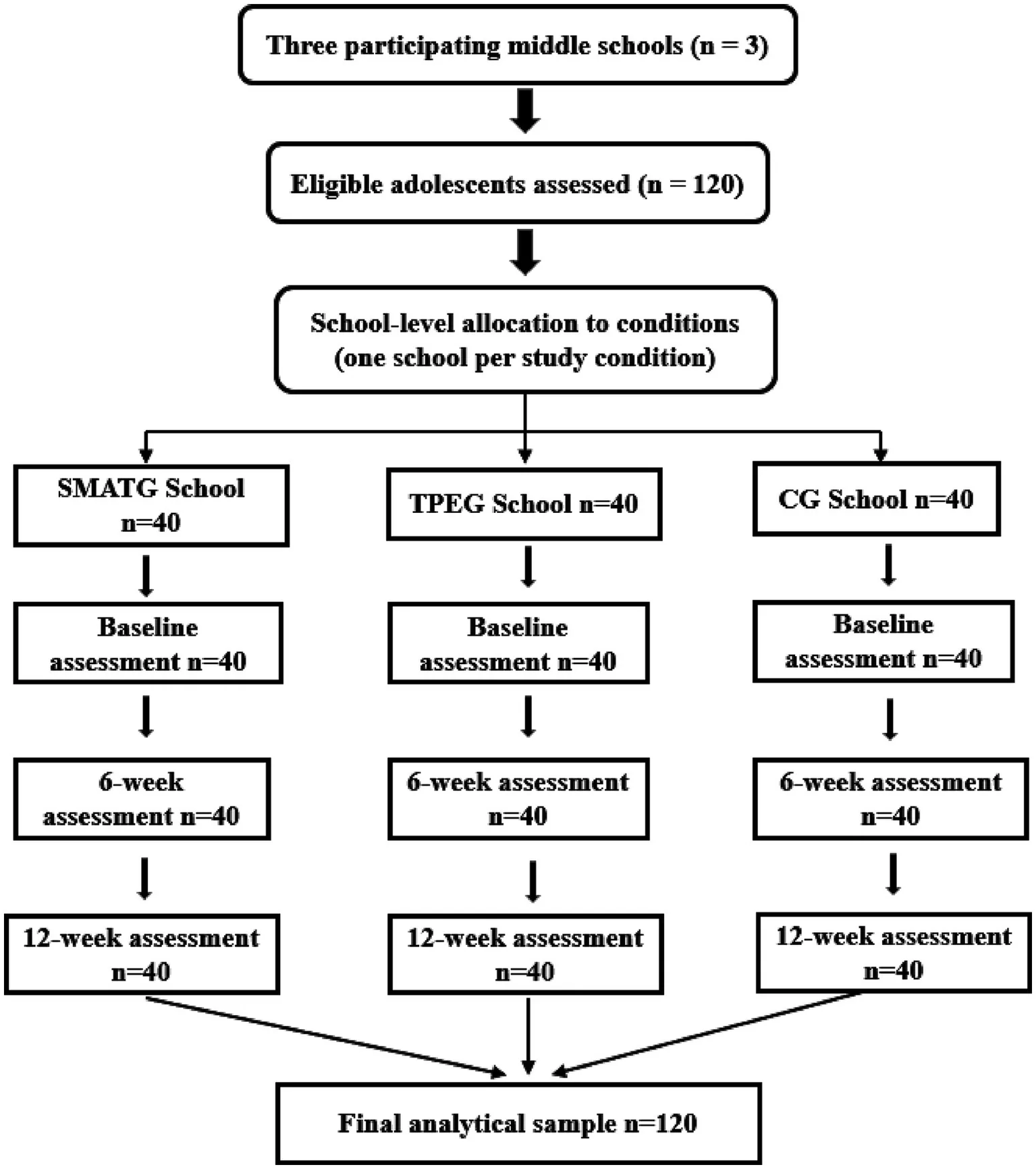
Participant flow and school-level allocation across the study conditions.

### Outcome measures

Outcome variables were categorized into three primary domains: physical fitness, executive function, and psychological resilience. All measurement instruments were selected based on their established psychometric properties, cross-cultural applicability, and widespread use in adolescent exercise science and educational research. Outcome variables were categorized into three domains: physical fitness, executive function, and psychological outcomes. All measures were selected based on their relevance to the multidimensional objectives of the intervention and their established use in adolescent exercise and educational research.

### Physical fitness variables

#### Agility

Agility was assessed using the Illinois Agility Test (IAT), a standardized field-based protocol designed to evaluate multidirectional speed, acceleration, deceleration, and change-of-direction ability. Participants completed the course on a regulation indoor non-slip surface under consistent environmental conditions. Completion time was electronically recorded using Brower Timing Systems TC-Timer photoelectric timing gates (Brower Timing Systems, Draper, UT, USA), ensuring precise, operator-independent measurement. The Illinois Agility Test has demonstrated excellent test–retest reliability in adolescent populations (ICC = 0.92–0.97), and has been validated as a sensitive indicator of multidirectional movement performance in youth sport and fitness research (Hachana et al., 2013; Saha et al., 2025).

#### Dynamic balance

Dynamic balance was evaluated using the Y-Balance Test (YBT), developed and commercialized by Functional Movement Systems (FMS) (Functional Movement Systems, Chatham, VA, USA). The YBT measures postural control and neuromuscular stability through single-leg stance reach tasks performed in three directions: anterior (ANT), posteromedial (PM), and posterolateral (PL). A composite reach score was calculated as a percentage of limb length to allow inter-individual comparisons. The Y-Balance Test demonstrates excellent intra-rater reliability (ICC = 0.85–0.91) and inter-rater reliability (ICC = 0.99–1.00), and has been validated for detecting lower-extremity neuromuscular adaptations in adolescent populations (Shaffer et al., 2013).

#### Muscular power

Lower-body explosive muscular power was assessed using the Standing Broad Jump Test (SBJT), also referred to as the Standing Long Jump Test. Participants performed a maximum-effort horizontal jump from a two-foot bilateral takeoff, landing on both feet. The best distance from three trials was recorded in centimeters using a BEURER PM 200 sports measurement mat (BEURER GmbH, Ulm, Germany) and verified with a calibrated steel measuring tape. The Standing Broad Jump Test demonstrates high test–retest reliability (ICC = 0.94–0.98) in school-aged populations and is widely recognized as a valid field measure of lower-extremity explosive power (Meylan et al., 2010; Prasad et al., 2025).

#### Cardiorespiratory endurance

Cardiorespiratory endurance was assessed using the 20-Metre Multistage Shuttle Run Test (20mMST), commonly referred to as the Beep Test or PACER Test, originally developed by Leger and Lambert (1982) and standardized for youth populations by Leger et al. (1988). The test protocol and audio signal were administered using the official FitnessGram® PACER audio CD (The Cooper Institute, Dallas, TX, USA). Participants ran continuously between two lines set 20 metres apart, synchronized with progressively increasing audio cues at one-minute stage intervals. The test was terminated when a participant failed to reach the line on two consecutive occasions. Cardiorespiratory fitness was assessed using the 20-Metre Multistage Shuttle Run Test (20mMST). Participants ran continuously between two lines positioned 20 m apart in synchrony with progressively increasing audio signals. The test was terminated when a participant failed to reach the required line on two consecutive occasions. Cardiorespiratory fitness was expressed as estimated peak oxygen uptake (estimated VO₂peak), calculated from the final running performance using the age-specific equation reported by Leger et al. (1988). Because oxygen consumption was estimated from field-test performance rather than measured directly through respiratory gas analysis, the resulting value was interpreted as estimated VO₂peak rather than directly measured Estimated VO₂peak. No laboratory-based gas-exchange measurement was performed. The 20mMST demonstrates excellent test–retest reliability (ICC = 0.89–0.97) and strong criterion validity against laboratory-measured Estimated VO₂peak in adolescents (r = 0.71–0.92) (Leger et al., 1988).

### Executive function variables

#### Inhibitory control

Inhibitory control was assessed using the Stroop Color and Word Test (SCWT), originally developed by Stroop (1935) and subsequently standardized by Golden (1978). The commercially published standardized version by PAR Inc. (Psychological Assessment Resources, Lutz, FL, USA) was administered in this study. The test consists of three conditions: (1) Word Reading, (2) Color Naming, and (3) Color-Word Interference. The interference score representing the difference between expected and actual performance on the color-word condition served as the primary index of inhibitory control and cognitive interference suppression. The SCWT demonstrates strong test–retest reliability (*r* = 0.73–0.89) and well-established construct validity as a measure of prefrontal inhibitory function in children and adolescents (Scarpina and Tagini, 2017; Strauss et al., 2006).

#### Working memory

Working memory capacity was evaluated using the Digit Span subtest of the Wechsler Intelligence Scale for Children Fifth Edition (WISC-V), developed and published by Pearson Clinical Assessment (Pearson Education Inc., San Antonio, TX, USA). The subtest includes three components: Digit Span Forward (verbal short-term memory), Digit Span Backward (working memory manipulation), and Digit Span Sequencing (mental reordering). Digit sequences were presented orally by a trained examiner at a rate of one digit per second, and total scaled scores were used for analysis. The Digit Span subtest demonstrates excellent internal consistency (α = 0.87–0.92) and test–retest reliability (*r* = 0.80–0.87), and is widely validated as a sensitive measure of phonological loop capacity and central executive function in adolescent populations (Alloway, 2010; Wechsler, 2024).

#### Cognitive flexibility

Cognitive flexibility was assessed using Trail Making Test Part B (TMT-B), sourced from the Delis-Kaplan Executive Function System (D-KEFS), developed and published by Pearson Clinical Assessment (Pearson Education Inc., San Antonio, TX, USA). In TMT-B, participants were required to draw lines connecting alternating numeric (1, 2, 3…) and alphabetic (A, B, C…) stimuli distributed across a standard A4 sheet as rapidly and accurately as possible. Total completion time (in seconds) served as the primary outcome measure, with lower scores indicating superior cognitive flexibility. The TMT-B demonstrates good test–retest reliability (ICC = 0.74–0.90) and strong criterion validity as a measure of mental set-shifting, visual scanning, and divided attention in adolescents (Arbuthnott and Frank, 2000; Delis et al., 2012).

The WISC-V Digit Span and D-KEFS TMT-B were administered individually by trained assessors in a quiet and standardized assessment setting within the participating schools. Participants were assessed sequentially to ensure consistent administration procedures across assessment sessions. The cognitive assessments were conducted according to the standardized procedures described for each instrument, with sufficient time provided between participants to minimize interruptions and maintain testing conditions.

### Psychological variables

#### Psychological resilience

Psychological resilience was assessed using the Connor-Davidson Resilience Scale (CD-RISC), developed by Connor and Davidson (2003) at Duke University Medical Center, USA. The 25-item full version of the scale was administered, measuring five empirically derived resilience dimensions: (1) personal competence and tenacity, (2) trust in one’s own instincts and tolerance of negative affect, (3) positive acceptance of change and secure relationships, (4) control, and (5) spiritual influences. Items are rated on a five-point Likert-type scale (0 = “not true at all” to 4 = “true nearly all the time”), yielding a total score ranging from 0 to 100, with higher scores indicating greater resilience. The CD-RISC demonstrates strong internal consistency (Cronbach’s α = 0.89), test–retest reliability (ICC = 0.87), and convergent and discriminant validity in adolescent and educational populations (Connor and Davidson, 2003; Yu and Zhang, 2007).

#### Self-regulation

Self-regulatory capacity was evaluated using the Adolescent Self-Regulatory Inventory (ASRI), developed by Moilanen (2007) at West Virginia University, USA. The ASRI is a 36-item self-report instrument measuring two superordinate dimensions of self-regulation: (1) short-term self-regulation (impulse control and behavioral inhibition) and (2) long-term self-regulation (goal pursuit, planning, and monitoring). Items are rated on a five-point Likert-type scale. The ASRI demonstrates acceptable to good internal consistency (Cronbach’s α = 0.77–0.84 for subscales), satisfactory test–retest reliability, and convergent validity with established measures of executive function and behavioral adjustment in adolescent samples (Moilanen, 2007; Moilanen et al., 2009).

#### Perceived stress

Perceived stress was measured using the 10-item Perceived Stress Scale (PSS-10), developed by Cohen et al. (1983) at Carnegie Mellon University, Pittsburgh, PA, USA. The PSS-10 is the most widely used validated instrument for assessing the subjective perception of stress in the past month, capturing the extent to which individuals appraise their life circumstances as unpredictable, uncontrollable, and overwhelming. Items are rated on a five-point Likert-type scale ranging from 0 (“never”) to 4 (“very often”), with positively worded items reverse-scored. Total scores range from 0 to 40, with higher scores indicating greater perceived stress. The PSS-10 demonstrates strong internal consistency (Cronbach’s α = 0.84–0.86), adequate test–retest reliability (r = 0.55–0.85), and well-established construct validity in adolescent psychological research (Cohen et al., 1983; Lee, 2012).

### Assessment procedure and testing sequence

Outcome assessments were conducted at baseline, 6 weeks, and 12 weeks using standardized procedures. Testing sessions were scheduled at approximately the same time of day (±1 h) across assessment points to minimize potential effects of circadian variation. Participants were instructed to avoid vigorous physical activity for at least 24 h before each assessment and to maintain their usual dietary and sleep routines.

At each assessment point, physical fitness, executive function, and psychological outcomes were assessed using a standardized testing sequence. Physical fitness assessments included the Illinois Agility Test, Y-Balance Test, Standing Broad Jump Test, and 20-Metre Multistage Shuttle Run Test. Executive function was assessed using the Stroop Color and Word Test, WISC-V Digit Span, and D-KEFS Trail Making Test Part B. Psychological questionnaires, including the Connor–Davidson Resilience Scale (CD-RISC), Adolescent Self-Regulatory Inventory (ASRI), and Perceived Stress Scale (PSS-10), were completed after the performance-based assessments.

The testing sequence was standardized across all three assessment points to minimize variation in measurement procedures. The physical assessments were conducted in a predetermined order, with standardized recovery intervals provided between physically demanding tests to minimize fatigue-related effects on subsequent performance. The 20-Metre Multistage Shuttle Run Test was completed before the cognitive assessments, with an adequate recovery period provided before commencement of cognitive testing. Psychological questionnaires were administered after completion of the performance-based assessments in a quiet and standardized setting.

The same testing environment, equipment, instructions, and assessment procedures were maintained across all measurement points. Assessors received training in the administration and scoring of the respective assessment instruments. However, assessors were not blinded to group allocation because of logistical constraints associated with the school-based intervention. To minimize potential measurement variability, standardized instructions and assessment procedures were used at each assessment point.

The mid- and post-intervention assessments were scheduled in relation to the intervention sessions to minimize the potential influence of acute exercise responses on outcome measurements. Participants were also instructed to maintain their usual routines regarding diet and sleep before each assessment.

### Safety and adverse events

Participant safety was monitored throughout the 12-week intervention period. Any exercise-related injury, adverse event, or health complaint occurring during or immediately after training sessions was recorded by the supervising staff and communicated to the research team. No intervention-related adverse events or serious exercise-related injuries were reported during the study period.

### Intervention protocol

The intervention was conducted over 12 weeks, with three sessions per week, and each session lasting 60 min, including 10 min of warm-up, 40 min of main training, and 10 min of cool-down. All sessions were supervised by certified martial arts instructors and physical education teachers with a minimum of 5 years of professional experience. Training intensity progressively increased according to the principle of progressive overload. The SMATG and TPEG sessions were delivered by different instructors, each with relevant professional qualifications and experience. All instructors followed standardized intervention protocols and session structures to ensure consistency in session content and delivery. However, because each study condition was implemented at a separate school, instructor-related effects could not be completely separated from the intervention condition and were therefore considered a potential source of confounding.

In Table 2 the school-Based Martial Arts Training Program was designed to improve multidimensional aspects of adolescent development, including physical fitness, executive function, and psychological resilience. The intervention incorporated progressive technical, neuromuscular, and cognitive demands adapted specifically for middle-school students within a safe educational environment. During the initial familiarization phase, participants focused on learning basic martial arts techniques and movement patterns to establish postural control, coordination, and exercise adherence. The neuromotor development phase emphasized dynamic balance, coordination, and reaction-based movement training. In the skill acquisition and cognitive-motor integration phases, greater emphasis was placed on attentional control, inhibitory response training, rapid decision-making, and movement sequencing through structured tactical drills and non-contact sparring activities. The final performance-oriented phases incorporated higher-intensity activities involving agility, tactical movement execution, reaction speed, and integrated motor-cognitive tasks. The progression in exercise complexity and intensity was systematically implemented to optimize physiological adaptation, cognitive stimulation, and psychological engagement while maintaining participant safety and adherence. The intervention structure was informed by previously published martial arts-based youth intervention studies demonstrating positive effects on executive function, self-regulation, motor coordination, and psychosocial development among adolescents.

**Table 2:** School-based martial arts training protocol (SMATG).

Phase	Weeks	Training focus	Training components	Intensity
Familiarization phase	Week 1–2	Fundamental movement adaptation	Basic stances, stepping patterns, posture alignment, flexibility drills, breathing control, basic punches and kicks	50%–60% HRmax
Neuromotor development phase	Week 3–4	Coordination and movement control	Movement sequencing, reaction drills, dynamic balance tasks, shadow practice, partner coordination drills (non-contact)	55%–65% HRmax
Skill acquisition phase	Week 5–6	Technical skill development	Combination techniques, agility-based movement drills, controlled striking patterns, attention-focus exercises, tactical footwork	60%–70% HRmax
Cognitive-motor integration phase	Week 7–8	Executive function enhancement	Dual-task drills, response inhibition activities, rapid decision-making tasks, controlled sparring drills, reaction training	65%–75% HRmax
Performance enhancement phase	Week 9–10	High-intensity motor execution	Advanced combinations, speed kicking drills, tactical movement patterns, agility circuits, partner-based reaction exercises	70%–80% HRmax
Performance consolidation phase	Week 11–12	Integrated physical and cognitive performance	Simulated martial arts routines, tactical sparring, high-intensity coordination tasks, maximal effort movement sequences	75%–85% HRmax

In Table 3 the Traditional Physical Education Program consisted of activities commonly implemented within standard school physical education curricula in China. The program emphasized general physical conditioning, aerobic fitness, flexibility, coordination, and recreational sport participation without inclusion of structured martial arts techniques or tactical combat-based training. Training intensity and exercise complexity progressively increased throughout the intervention period. Activities included jogging, bodyweight conditioning exercises, aerobic circuits, flexibility routines, agility drills, and team sport participation. The program was designed to match the frequency and duration of the martial arts intervention while maintaining equivalent supervision and training exposure.

**Table 3:** Traditional physical education training protocol (TPEG).

Phase	Weeks	Training focus	Training components	Intensity
Familiarization Phase	Week 1–2	General physical adaptation	Light jogging, stretching exercises, mobility drills, recreational movement activities	50%–60% HRmax
Fundamental Fitness Phase	Week 3–4	Basic fitness development	Calisthenics, aerobic exercises, flexibility training, low-intensity coordination drills	55%–65% HRmax
Physical conditioning phase	Week 5–6	Cardiovascular and muscular conditioning	Circuit training, running drills, bodyweight exercises, endurance-based activities	60%–70% HRmax
Motor Skill development phase	Week 7–8	Agility and coordination improvement	Agility ladder drills, team sport activities, dynamic balance tasks, relay exercises	65%–75% HRmax
Performance conditioning phase	Week 9–10	Higher-intensity physical activity	Moderate-to-high intensity games, aerobic conditioning, plyometric activities	70%–80% HRmax
Performance maintenance phase	Week 11–12	Integrated physical performance	Mixed physical education activities, conditioning drills, team-based sport participation	75%–85% HRmax

Participants assigned to the Control Group (CG) did not receive the additional structured martial arts or traditional physical education intervention provided to the two intervention groups. However, participants continued their regular school curriculum, including scheduled physical education lessons as part of the standard school program. The frequency, duration, and general content of these routine physical education lessons were documented from school timetables and school records. Participants were instructed not to participate in additional organized martial arts or structured exercise programs outside school during the intervention period. Weekly communication with school staff and participant self-report were used to identify potential contamination. Accordingly, the CG should be interpreted as a usual-activity control group rather than a completely sedentary or passive control condition.

### Statistical analysis

Statistical analyses were performed using IBM SPSS Statistics version 26. Continuous variables were summarized as mean ± standard deviation (SD). Distributional assumptions were assessed using the Shapiro–Wilk test, and homogeneity of variance was evaluated using Levene’s test. A mixed multivariate analysis of variance (MANOVA) was initially conducted to examine the effects of Group (SMATG, TPEG, and CG), Time (baseline, 6 weeks, and 12 weeks), and the Group × Time interaction across the multidimensional outcome set. Significant multivariate effects were followed, where appropriate, by univariate repeated-measures analyses for individual outcomes to identify outcome-specific Group × Time interactions. When the assumption of sphericity was violated, the Greenhouse–Geisser correction was applied. Significant effects were examined using Bonferroni-adjusted pairwise comparisons. Partial eta squared (η^2^p) was reported as the effect-size measure, and statistical significance was set at *p* < 0.05. All 120 participants were included in the analyses at each assessment point. No missing outcome observations were identified; therefore, no imputation was required. Given the school-level allocation, with each study condition implemented in a separate school, the statistical findings were interpreted as associations between study condition and longitudinal outcome changes rather than as unbiased estimates of an individual-level causal intervention effect.

## Results

Table 4 presents the descriptive statistics (Mean ± Standard Deviation) for all physical fitness, executive function, and psychological variables measured across the three assessment periods (pre-test, 6 weeks, and 12 weeks) among the school-Based Martial Arts Training Group (SMATG), Traditional Physical Education Group (TPEG), and Control Group (CG). At baseline, all groups demonstrated comparable values across all dependent variables, indicating initial group homogeneity prior to the intervention. Across the 12-week observation period, outcome trajectories differed among the three study conditions. The SMATG generally showed greater improvements in physical fitness, executive function, and psychological outcomes than the TPEG and CG. The TPEG demonstrated improvements in several outcomes, whereas changes in the CG were comparatively smaller. Detailed descriptive statistics for each outcome at baseline, 6 weeks, and 12 weeks are presented in Table 4, while inferential analyses of Group × Time interactions are presented in Table 6. Specifically, significant reductions were observed in agility time, Stroop interference score, Trail Making Test-B completion time, and perceived stress levels, while marked increases were identified in dynamic balance, standing broad jump performance, Estimated VO₂peak, digit span score, psychological resilience, and self-regulation. The TPEG demonstrated moderate improvements across several variables; however, the magnitude of improvement remained consistently smaller than that observed in the SMATG. In contrast, the CG exhibited minimal or negligible changes across all variables throughout the intervention period.

**Table 4:** Mean ± SD values of physical fitness, executive function, and psychological variables across groups and time points.

Variables	Group	Pre-test (M ± SD)	6 Weeks (M ± SD)	12 Weeks (M ± SD)
Agility (sec)	SMATG	19.82 ± 1.14	18.74 ± 1.02	17.58 ± 0.95
	TPEG	19.77 ± 1.11	19.21 ± 1.05	18.63 ± 1.01
	CG	19.69 ± 1.09	19.61 ± 1.08	19.54 ± 1.06
Dynamic balance (%)	SMATG	84.35 ± 5.22	89.46 ± 4.91	94.71 ± 4.63
	TPEG	84.12 ± 5.18	86.21 ± 5.01	88.63 ± 4.88
	CG	84.28 ± 5.31	84.41 ± 5.26	84.56 ± 5.20
Standing broad jump (cm)	SMATG	162.84 ± 12.55	171.42 ± 11.87	181.63 ± 11.12
	TPEG	163.11 ± 12.48	167.24 ± 12.03	171.82 ± 11.74
	CG	162.95 ± 12.61	163.36 ± 12.44	163.81 ± 12.26
Estimated VO₂peak (ml·kg^−1^·min^−1^)	SMATG	39.62 ± 3.44	42.11 ± 3.18	45.34 ± 3.06
	TPEG	39.71 ± 3.38	40.96 ± 3.21	42.47 ± 3.10
	CG	39.58 ± 3.41	39.66 ± 3.37	39.74 ± 3.34
Inhibitory control	SMATG	47.83 ± 5.18	42.74 ± 4.87	37.62 ± 4.44
	TPEG	47.71 ± 5.10	45.82 ± 4.94	43.66 ± 4.72
	CG	47.94 ± 5.23	47.63 ± 5.15	47.21 ± 5.11
Working memory	SMATG	8.42 ± 1.13	9.56 ± 1.06	10.84 ± 0.98
	TPEG	8.38 ± 1.10	8.91 ± 1.04	9.41 ± 1.01
	CG	8.40 ± 1.11	8.46 ± 1.09	8.51 ± 1.08
Cognitive flexibility	SMATG	78.42 ± 8.91	71.16 ± 8.02	63.38 ± 7.44
	TPEG	78.67 ± 8.84	75.24 ± 8.31	72.43 ± 7.96
	CG	78.51 ± 8.88	78.22 ± 8.74	77.94 ± 8.69
Psychological resilience	SMATG	61.84 ± 6.43	69.22 ± 6.01	77.31 ± 5.54
	TPEG	62.03 ± 6.36	65.17 ± 6.08	68.42 ± 5.91
	CG	61.92 ± 6.40	62.11 ± 6.35	62.26 ± 6.29
Self-regulation score	SMATG	108.42 ± 9.24	116.81 ± 8.77	126.74 ± 8.16
	TPEG	108.63 ± 9.18	112.06 ± 8.91	115.42 ± 8.63
	CG	108.54 ± 9.20	108.81 ± 9.14	109.03 ± 9.06
Perceived stress	SMATG	24.82 ± 3.84	20.63 ± 3.47	16.74 ± 3.12
	TPEG	24.67 ± 3.79	22.93 ± 3.58	21.16 ± 3.41
	CG	24.74 ± 3.81	24.58 ± 3.76	24.43 ± 3.72

Table 5 presents the results of the Mixed Multivariate Analysis of Variance (Mixed MANOVA) conducted to evaluate the multivariate effects of Group, Time, and Group × Time interaction across the combined dependent variables. The analysis revealed statistically significant main effects for Group (Wilks’ Lambda = 0.328, *F* = 7.86, *p* < 0.001, partial η^2^ = 0.42) and Time (Wilks’ Lambda = 0.194, *F* = 18.54, *p* < 0.001, partial η^2^ = 0.63), indicating overall differences among groups and across assessment periods. Furthermore, a significant Group × Time interaction effect was identified (Wilks’ Lambda = 0.214, *F* = 6.84, *p* < 0.001, partial η^2^ = 0.41), demonstrating that the pattern of change over time differed significantly among the three groups. The observed partial eta squared values indicated large multivariate effect sizes, reflecting substantial differences in longitudinal outcome patterns across the study conditions.

**Table 5:** Mixed MANOVA results for multidimensional outcome variables.

Effect	Wilks’ Lambda	F-value	Hypothesis df	Error df	*p*-value	Partial η^2^
Group	0.328	7.86	20	216	<0.001	0.42
Time	0.194	18.54	20	216	<0.001	0.63
Group × Time	0.214	6.84	40	432	<0.001	0.41

Table 6 summarizes the repeated-measures ANOVA results examining Group × Time interaction effects for all physical fitness, executive function, and psychological variables. Significant interaction effects were identified across all measured outcomes (*p* < 0.001), indicating that the magnitude and direction of changes over time differed significantly among the intervention groups. Within the physical fitness domain, significant improvements were observed for agility, dynamic balance, standing broad jump performance, and Estimated VO₂peak, with the SMATG demonstrating the greatest magnitude of change compared with the TPEG and CG. Similarly, significant interaction effects were identified for executive function variables, including Stroop interference, digit span score, and Trail Making Test-B performance, suggesting enhanced inhibitory control, working memory, and cognitive flexibility following the martial arts intervention. In addition, significant interaction effects were observed for psychological resilience, self-regulation, and perceived stress, indicating meaningful psychosocial adaptations among participants in the SMATG. The partial eta squared values ranged from 0.29 to 0.39, representing moderate-to-large effect sizes across the multidimensional outcomes. All omnibus Group × Time interactions reached statistical significance (*p* < 0.001), whereas the magnitude of significance varied across the Bonferroni-adjusted pairwise comparisons.

**Table 6:** Group × time interaction effects for physical fitness, executive function, and psychological outcomes.

Domain	Variable	Source	F-value	*p-value*	Partial η^2^
Physical Fitness	Agility	Group × Time	24.81	<0.001	0.39
	Dynamic balance	Group × Time	21.44	<0.001	0.36
	Standing broad jump	Group × Time	19.63	<0.001	0.33
	Estimated VO₂peak	Group × Time	17.88	<0.001	0.30
Executive Function	Inhibitory control	Group × Time	22.73	<0.001	0.37
	Working memory	Group × Time	16.92	<0.001	0.29
	Cognitive flexibility	Group × Time	20.18	<0.001	0.34
Psychological Variables	Psychological resilience	Group × Time	23.66	<0.001	0.38
	Self-regulation	Group × Time	18.27	<0.001	0.31
	Perceived stress	Group × Time	21.95	<0.001	0.36

Table 7 presents the Bonferroni-adjusted *post hoc* pairwise comparisons conducted at post-test to determine specific between-group differences following the 12-week intervention period. Significant differences were observed between the school-Based Martial Arts Training Group (SMATG) and both the Traditional Physical Education Group (TPEG) and Control Group (CG) across all physical fitness, executive function, and psychological variables. Within the physical fitness domain, the SMATG demonstrated significantly superior agility performance, dynamic balance, standing broad jump distance, and Estimated VO₂peak compared with the other groups. Similarly, participants in the SMATG exhibited significantly lower Stroop interference scores and Trail Making Test-B completion times, accompanied by significantly higher digit span scores, indicating enhanced executive functioning. In the psychological domain, significantly greater improvements in psychological resilience and self-regulation, together with significantly lower perceived stress scores, were observed in the SMATG compared with both TPEG and CG. Although the TPEG also demonstrated significantly better outcomes than the CG for several variables, the magnitude of improvement remained consistently smaller than that observed in the SMATG. Collectively, these findings support the superior effectiveness of the school-based martial arts intervention in promoting multidimensional physical, cognitive, and psychological development among adolescents.

**Table 7:** Bonferroni-adjusted pairwise comparisons between groups at post-test for outcomes showing significant group × time interactions.

Domain	Variable	Comparison	MD	SE	*p*-value
Physical fitness	Agility	SMATG vs. TPEG	−1.05	0.22	<0.001
		SMATG vs. CG	−1.96	0.23	<0.001
		TPEG vs. CG	−0.91	0.21	0.002
	Dynamic balance	SMATG vs. TPEG	6.08	1.02	<0.001
		SMATG vs. CG	10.15	1.06	<0.001
		TPEG vs. CG	4.07	0.98	0.004
	Standing broad jump	SMATG vs. TPEG	9.81	2.41	0.003
		SMATG vs. CG	17.82	2.53	<0.001
		TPEG vs. CG	8.01	2.37	0.011
	Estimated VO₂peak	SMATG vs. TPEG	2.87	0.64	<0.001
		SMATG vs. CG	5.60	0.68	<0.001
		TPEG vs. CG	2.73	0.61	<0.001
Executive function	Inhibitory control	SMATG vs. TPEG	−6.04	1.11	<0.001
		SMATG vs. CG	−9.59	1.16	<0.001
		TPEG vs. CG	−3.55	1.08	0.014
	Working memory	SMATG vs. TPEG	1.43	0.24	<0.001
		SMATG vs. CG	2.33	0.26	<0.001
		TPEG vs. CG	0.90	0.22	0.003
	Cognitive flexibility	SMATG vs. TPEG	−9.05	1.87	<0.001
		SMATG vs. CG	−14.56	1.95	<0.001
		TPEG vs. CG	−5.51	1.81	0.018
Psychological variables	Psychological resilience	SMATG vs. TPEG	8.89	1.26	<0.001
		SMATG vs. CG	15.05	1.31	<0.001
		TPEG vs. CG	6.16	1.22	<0.001
	Self-regulation	SMATG vs. TPEG	11.32	2.14	<0.001
		SMATG vs. CG	17.71	2.21	<0.001
		TPEG vs. CG	6.39	2.06	0.016
	Perceived stress	SMATG vs. TPEG	−4.42	0.71	<0.001
		SMATG vs. CG	−7.69	0.75	<0.001
		TPEG vs. CG	−3.27	0.69	0.001

## Discussion

The present quasi-experimental study examined the multidimensional effects of a 12-week school-based martial arts intervention on physical fitness, executive function, and psychological outcomes in adolescents. The findings demonstrated that the school-Based Martial Arts Training Group (SMATG) achieved significantly superior improvements across all 10 measured variables compared with both the Traditional Physical Education Group (TPEG) and the Control Group (CG), with moderate-to-large effect sizes (partial η^2^ = 0.29–0.39). The present quasi-experimental study examined the multidimensional effects of a 12-week school-based martial arts intervention on physical fitness, executive function, and psychological outcomes in adolescents. The school-Based Martial Arts Training Group (SMATG) demonstrated significantly greater improvements across all 10 measured variables compared with the Traditional Physical Education Group (TPEG) and Control Group (CG), with moderate-to-large effect sizes (partial η^2^ = 0.29–0.39). The Mixed MANOVA also indicated a significant Group × Time interaction (Wilks’ Λ = 0.214, partial η^2^ = 0.41). Collectively, these findings suggest that structured martial arts training may be associated with improvements across multiple domains of adolescent development. However, given the quasi-experimental design and school-level allocation, these differences should be interpreted cautiously and should not be regarded as definitive evidence of individual-level causal effects.

The SMATG demonstrated progressive reductions in agility completion time from 19.82 ± 1.14 s at baseline to 17.58 ± 0.95 s at 12 weeks, substantially outperforming both the TPEG and CG. This finding aligns with evidence from Shen (2024), who reported that combined balance and plyometric training in female adolescent taekwondo athletes produced significant improvements in direction-change speed and dynamic postural stability indices, attributing these adaptations to the neuromuscular demands of martial arts-specific footwork and rapid limb coordination. The multidirectional movement patterns inherent to martial arts, including evasive stepping, pivoting, and rapid stance transitions, closely mirror the motor demands of standard agility assessments. A systematic review by Stamenković et al. (2022) further confirmed that martial arts programmes in school-aged children consistently enhanced agility and motor coordination through sustained improvements in sensorimotor integration and reaction time. The comparatively modest agility gains observed in the TPEG may reflect differences in the reactive and multidirectional neuromuscular demands of the respective physical education activities.

Dynamic balance improved markedly in the SMATG, rising from 84.35 ± 5.22% at pre-test to 94.71 ± 4.63% at 12 weeks. This magnitude of improvement is consistent with recent evidence from Suarez-Villadat et al. (2025), who found that a 24-week adapted judo programme significantly enhanced dynamic balance and proprioceptive control in adolescents, attributing the adaptations to the constant multi-planar weight-shifting and controlled falling sequences that are central to grappling-based martial arts practice. Similarly, Rodrigues et al. (2024) concluded in a decade-long scoping review of 102 studies across 28 countries that martial arts and combat sports interventions are among the most consistently effective modalities for promoting balance and motor competence in youth, irrespective of the specific martial art practiced. The greater balance improvements observed in the SMATG may be partly related to the proprioceptive and multidirectional movement demands associated with partner-dependent martial arts practice.

Explosive lower-limb power, assessed via the standing broad jump, improved substantially in the SMATG from 162.84 ± 12.55 cm to 181.63 ± 11.12 cm over 12 weeks. These gains are consistent with findings reported by Roh et al. (2020), who demonstrated that regular taekwondo training significantly improved Sargent jump performance in overweight adolescents, attributing explosive power gains to the repeated plyometric loading of kicking techniques and dynamic stance work. Kabadayı et al. (2022) similarly observed significant improvements in lower-body power among youth karate athletes following a 12-week core training intervention, highlighting that the multi-planar explosive demands of striking martial arts generate progressive overload on fast-twitch musculature sufficient to elicit meaningful power adaptations. The biomechanical specificity of techniques such as front kicks, roundhouse kicks, and takedown entries, each demanding rapid, coordinated triple extension of the lower limb, closely parallels the movement mechanics of horizontal jump performance, providing a plausible explanation for the greater improvements observed in the SMATG.

Estimated cardiorespiratory fitness, expressed as estimated VO₂peak, improved from 39.62 ± 3.44 to 45.34 ± 3.06 mL·kg^−1^·min^−1^ in the SMATG, representing a 14.4% increase over 12 weeks. Ciccotti Saraiva et al. (2022) reported that 9 months of judo or Muay Thai practice significantly increased Estimated VO₂peak in children and adolescents, demonstrating that sustained martial arts participation provides sufficient aerobic stimulus for cardiorespiratory adaptation even at twice-weekly training frequencies. Adanan et al. (2024) further documented that adolescent taekwondo and wushu practitioners exhibited significantly higher aerobic capacity profiles compared with sedentary peers, reflecting the chronic cardiovascular adaptations associated with intermittent high-intensity martial arts training. The intermittent, high-intensity nature of martial arts, characterised by bursts of explosive effort interspersed with brief active recovery, closely mirrors the physiological stimulus of high-intensity interval training (HIIT), which Crowley et al. (2022) identified as among the most potent promoters of Estimated VO₂peak across exercise modalities. These converging lines of evidence substantiate the cardiorespiratory benefits observed in the present SMATG and suggesting that the intensity and intermittent nature of structured martial arts training may provide a different cardiorespiratory stimulus than the activities undertaken in the TPEG (Crowley et al., 2022).

The SMATG exhibited substantial reductions in Stroop interference scores from 47.83 ± 5.18 to 37.62 ± 4.44, indicating marked gains in inhibitory control. These findings are consistent with evidence from Harwood-Gross et al. (2021), who documented significant improvements in inhibition and cognitive shifting among at-risk adolescent boys following 6 months of martial arts training, identifying oxytocin reactivity as a key biological mediator of post-intervention cognitive change. Wang and Bei (2024) demonstrated, in a 12-week randomised controlled trial using EEG, that Tai Chi training significantly improved Stroop accuracy and reduced reaction time under incongruent conditions in college students, with concurrent modulation of frontal alpha and theta band activity providing neurophysiological evidence that mind–body martial arts practice may contribute to adaptations in neural processes supporting inhibitory control. The demanding attentional requirements of martial arts, requiring practitioners to simultaneously anticipate opponent actions, execute precise techniques, and suppress habitual motor responses under time pressure, provide a naturalistic training environment for inhibitory control that directly parallels the cognitive architecture measured by the Stroop paradigm. Liu L. et al. (2025), in a recent systematic review and meta-analysis of 21 randomised controlled trials, confirmed that physical exercise interventions produce significant improvements in inhibitory control in adolescents, with open-skill activities generating the largest effect sizes due to their greater unpredictability and cognitive engagement demands.

Working memory capacity, assessed via the digit span task, improved significantly in the SMATG from 8.42 ± 1.13 to 10.84 ± 0.98 over 12 weeks. Song et al. (2023) demonstrated in a meta-analysis that physical activity interventions elicited significant improvements in working memory in children and adolescents (SMD = −0.50), with exercise programmes exceeding three sessions per week producing medium-to-high effect sizes specifically for working memory outcomes. The association between martial arts practice and working memory is further supported by cross-sectional evidence from Kaddouri et al. (2026), who reported that children practicing full-contact and kung fu demonstrated superior digit span and Corsi block-tapping scores compared with non-practising peers, attributing these differences to the sequential motor planning and tactical recall demands intrinsic to martial arts training. Mechanistically, the need to encode, retain, and update complex movement sequences, competitive strategies, and opponent behavioral patterns during martial arts training constitutes a chronic working memory load that may place repeated demands on neural processes supporting working memory, regions critically implicated in verbal and visuospatial working memory capacity (Guo et al., 2025).

Cognitive flexibility, indexed by TMT-B completion time, improved substantially in the SMATG from 78.42 ± 8.91 s to 63.38 ± 7.44 s, representing a 19.2% reduction. Alexandrino et al. (2026) demonstrated in a cluster randomised trial that a six-week school-based HIIT programme significantly reduced TMT-B completion times in adolescents aged 15–17 years, confirming the sensitivity of set-shifting capacity to structured vigorous exercise within educational settings. The enriched sport programme study by Gentile et al. (2020) further observed significant TMT-B improvements alongside digit span backward gains in children receiving cognitively enriched physical activity, suggesting that interventions imposing concurrent motor and cognitive demands are particularly effective in promoting cognitive flexibility. Critically, martial arts training may exert stronger effects on TMT-B performance than generic exercise due to the continuous requirement to switch attentional focus between offensive and defensive tasks, adapt movement strategies in real time, and suppress prepotent responses under competitive pressure, all of which map directly onto the set-shifting and task-alternation demands of TMT-B. This interpretation is supported by a meta-analysis by Liu C. et al. (2025), which reported that physical activity interventions produced significant improvements in cognitive flexibility in children and adolescents with neurodevelopmental profiles (Hedges’ g = 0.60), with open-skill, unpredictable exercise modalities demonstrating the greatest effect magnitudes.

Psychological resilience, measured via the CD-RISC, improved markedly in the SMATG from 61.84 ± 6.43 to 77.31 ± 5.54 over 12 weeks, a 25.0% increase that substantially exceeded gains in the TPEG and CG. Moore et al. (2021) provided direct experimental support for this outcome, demonstrating in a randomised controlled trial that school-based martial arts training significantly enhanced resilience in secondary school students, conceptualising the structured adversity, progressive mastery, and goal-setting culture of martial arts as key mechanisms underlying resilience development. Li X. et al. (2024) further established, through a serial mediation model, that physical activity improves adolescent resilience via sequential pathways through self-efficacy and basic psychological needs satisfaction, constructs that are particularly well-cultivated within the hierarchical belt and rank progression systems characteristic of martial arts training. A systematic review and meta-analysis by Cai et al. (2025), encompassing randomised controlled trials of school-based interventions across multiple countries, confirmed that structured school-based physical and psychological programmes yield significant resilience improvements in children and adolescents, with effect sizes augmented when interventions incorporate disciplined practice, goal-setting, and interpersonal challenge, precisely the features that distinguish martial arts from conventional physical education. The relatively small changes observed in the CG suggest that the structured training conditions may have provided additional opportunities for mastery, goal-setting, and interpersonal engagement.

Self-regulation scores improved substantially in the SMATG from 108.42 ± 9.24 to 126.74 ± 8.16, representing a 16.9% gain versus negligible change in the CG. Yu and Mantuhac (2024) demonstrated, in a study examining martial arts integration within physical education curricula, that martial arts education exerted a significant positive effect on student self-regulation across behavioral, cognitive, and emotional domains, underscoring the pedagogical value of martial arts’ inherent emphasis on discipline, deferred gratification, and rule-governed conduct. Kabadayı et al. (2022) identified that the structured, code-governed nature of martial arts training, encompassing respect rituals, formal instruction hierarchies, and progressive competency standards, may provide repeated opportunities to practice self-regulation within structured behavioral, cognitive, and social contexts. Collectively, these findings suggest that the self-regulation benefits of martial arts derive not merely from physical exertion but from the socio-pedagogical architecture of martial arts training as a disciplinary practice.

Perceived stress, assessed via the PSS-10, declined significantly in the SMATG from 24.82 ± 3.84 to 16.74 ± 3.12, a 32.6% reduction that substantially exceeded decreases in the TPEG (14.2%) and was virtually absent in the CG. Lee and Ihn (2023) identified martial arts as a potentially useful approach for addressing stress among adolescents, noting that the physical exertion, structured mindfulness, and community belonging afforded by martial arts training address multiple simultaneous stress pathways unavailable in passive stress management approaches. Yıldız (2022) similarly demonstrated that karate practice produced significant reductions in perceived stress and corresponding improvements in self-confidence among sedentary teenagers, with effect magnitudes exceeding those typically reported for general aerobic exercise interventions. The stress-reducing mechanisms associated with martial arts may be multifactorial: physiological pathways include exercise-induced reductions in hypothalamic–pituitary–adrenal axis reactivity and cortisol habituation, while psychological pathways include enhanced self-efficacy derived from progressive skill mastery, improved emotion regulation capacity, and the social support embedded within the dojo training community (Harwood-Gross et al., 2021; Cai et al., 2025). Furthermore, proposed a PERMA-aligned framework for understanding the stress-buffering properties of martial arts, in which positive emotion, engagement, relationships, meaning, and accomplishment, each cultivated through martial arts practice, collectively attenuate perceived stress responses (Lee and Ihn, 2023). The present findings suggest that participation in the structured martial arts condition was associated with reductions in perceived stress over the 12-week intervention period. However, the quasi-experimental design and school-level allocation prevent firm conclusions regarding whether these changes were attributable specifically to martial arts training.

Across the 10 outcome variables, the SMATG demonstrated greater improvements than the TPEG, despite both intervention groups receiving comparable scheduled physical activity time. This pattern suggests that differences in the structure, content, and specific demands of the training activities may have contributed to the observed differential responses. The TPEG also demonstrated improvements across several outcomes, indicating that conventional physical education may provide beneficial effects, although the magnitude of change was generally smaller than that observed in the SMATG. These findings highlight the potential value of incorporating structured, cognitively engaging, and progressively organized activities into school-based physical education. Nevertheless, because the study conditions were implemented at the school level, alternative explanations related to school-specific contextual factors cannot be excluded.

The present study possesses several methodological strengths, including its multidimensional assessment framework integrating physical fitness, executive function, and psychological outcomes within a single school-based intervention. The repeated-measures design, inclusion of both traditional physical education and control conditions, standardized intervention procedures, and use of validated outcome measures enhanced the consistency and practical relevance of the findings. Furthermore, the study was conducted within a real-world educational environment, thereby supporting the ecological relevance and potential applicability of school-based martial arts programmes.

Nevertheless, several limitations should be acknowledged. First, an important limitation concerns the school-level allocation of participants. Because each study condition was implemented in a separate school, school and intervention condition were inherently confounded. Consequently, observed differences between groups may partly reflect school-specific contextual characteristics rather than the intervention itself. Although measured baseline demographic characteristics were comparable across groups, similarity in observed characteristics cannot exclude unmeasured differences between schools. Future studies should recruit multiple schools per intervention condition and, where feasible, randomize schools or classes to study conditions to better distinguish intervention-related effects from school-level contextual influences.

Second, the absence of long-term follow-up assessments prevented evaluation of whether the observed changes in physical, cognitive, and psychological outcomes were sustained beyond the 12-week intervention period. Third, the study included only male adolescents; therefore, the findings may not be generalizable to female adolescents, and potential sex-specific responses could not be examined. In addition, biological maturation status was not assessed or separately analyzed, although developmental differences during adolescence may influence physical fitness, cognitive performance, and psychological outcomes. Fourth, outcome assessors were not blinded to group allocation, which may have introduced measurement bias, particularly for outcomes involving self-reported psychological measures.

Fifth, estimated VO₂peak was derived from the 20-m shuttle run rather than directly measured using respiratory gas analysis and should therefore be interpreted as an estimate of cardiorespiratory fitness rather than as a directly measured value. The estimated VO₂peak values were also expressed using conventional body-mass ratio scaling. Given the potential influence of growth and maturation on body size during adolescence, future studies should consider allometric scaling or other appropriate approaches to body-size adjustment when examining cardiorespiratory fitness. Sixth, although the Y-Balance Test assesses anterior, posteromedial, and posterolateral reach directions, the present dataset contained only the composite normalized reach score; therefore, directional adaptations could not be examined separately. Finally, participants were recruited from middle schools within a single geographical region of China, which may limit the generalizability of the findings to other cultural, educational, and demographic populations.

Future research should employ randomized controlled designs involving multiple schools per intervention condition, with randomization at the school or class level where appropriate, to reduce school-level confounding and strengthen causal inference. Longer follow-up periods should also be incorporated to examine the persistence of observed changes. Studies including both male and female adolescents should assess potential sex-specific responses, while systematic assessment of biological maturation should be incorporated to account for developmental variability. Where feasible, direct measurement of cardiorespiratory fitness, appropriate body-size scaling, and blinded outcome assessment should be used to strengthen measurement validity. Further research incorporating neurophysiological and other objective assessment techniques may help clarify the mechanisms potentially underlying changes in executive function and psychological outcomes associated with school-based martial arts training.

## Conclusion

The present quasi-experimental study found that adolescents participating in a structured 12-week school-based martial arts training condition demonstrated greater improvements across measures of physical fitness, executive function, psychological resilience, self-regulation, and perceived stress than those in the Traditional Physical Education Group and Control Group. These findings suggest that structured martial arts training may be a promising approach for supporting multiple aspects of adolescent development within school-based physical education. The observed improvements, particularly across multiple outcome domains, highlight the potential value of incorporating cognitively engaging and progressively structured physical activities into school programmes. However, the findings should be interpreted cautiously because the study used school-level allocation without individual randomization, with one school implementing each study condition. Consequently, school-specific contextual factors may have contributed to the observed between-group differences, and the findings cannot be interpreted as definitive evidence of an intervention-specific causal effect. Future research should therefore involve multiple schools per study condition, preferably with randomized allocation at the school or class level, together with longer follow-up periods, assessment of biological maturation and sex-specific responses, and blinded outcome assessment where feasible. Such studies would help determine the reproducibility, durability, and intervention-specific nature of the observed effects.

## Data Availability

The original contributions presented in the study are included in the article/supplementary material, further inquiries can be directed to the corresponding author.
